# REPLY TO THE AUTHORS: Re: One-day voiding diary in the evaluation of Lower Urinary Tract Symptoms in children

**DOI:** 10.1590/S1677-5538.IBJU.2022.0059.1

**Published:** 2023-04-05

**Authors:** Hanny Helena Masson Franck, Ana Carolina S. Guedes, Yago Felyppe S. Alvim, Thamires M. S. de Andrade, Liliana Fajardo Oliveira, Lidyanne Ilidia da Silva, André Avarese de Figueiredo, José de Bessa, José Murillo B. Netto

**Affiliations:** 1 Departamento de Cirurgia Faculdade de Medicina Universidade Federal de Juiz de Fora Juiz de Fora MG Brasil Departamento de Cirurgia da Faculdade de Medicina – Universidade Federal de Juiz de Fora (UFJF), Juiz de Fora, MG, Brasil;; 2 Escola de Enfermagem Universidade Federal de Juiz de Fora Juiz de Fora MG Brasil Escola de Enfermagem – Universidade Federal de Juiz de Fora (UFJF),Juiz de Fora, MG, Brasil;; 3 Escola de Fisioterapia Faculdade de Ciências Médicas e da Saúde de Juiz de Fora Juiz de Fora MG Brasil Escola de Fisioterapia – Faculdade de Ciências Médicas e da Saúde de Juiz de Fora (HMTJ/SUPREMA), Juiz de Fora, MG, Brasil;; 4 Departamento de Cirurgia Faculdade de Medicina Universidade Estadual de Feira de Santana Feira de Santana BA Brasil Departamento de Cirurgia da Faculdade de Medicina – Universidade Estadual de Feira de Santana (UEFS), Feira de Santana, BA, Brasil

To the editor,

We are thankful for the comments and agree with the delicate considerations (1).

Repeated measures of clinical parameters increase accuracy, identify possible variations, and minimize measurement bias. Our study demonstrates, despite possible biases, that there is a good correlation between the two formats (2).

The 3-day voiding diary is the “Gold standard” in assessing LUTS in children. Difficulties in obtaining adequate assessments, especially in more complex cases and families with low literacy, have motivated other authors and our group to search for simplified alternatives.

Our proposal would minimize patient/caregiver burden and increase the rate of complete responses.

Other authors have studied these aspects previously. Elmer et al. evaluated incontinent women and showed promising results with this approach (3). In the same direction, Veiga et al. demonstrated a good correlation between the two formats and considered that a 2-day bladder diary was sufficient to evaluate bladder capacity and fluid intake (4).

Our findings reinforce this idea that a simplified version could be an attractive alternative.

Furthermore, we plan to evaluate asymptomatic and non-neurotypical children. The difficulties in investigating asymptomatic children (ethical aspects and little cooperation from parents) are important limiting factors.

Further studies are needed to validate the one-day voiding diary in evaluating LUTS and clarify the accurate correlation between objectives bladder parameters (Maximum Voided Volume) and estimated bladder capacity (EBC) in the asymptomatic and children with LUTS.

The authors.
